# Serum Homocysteine Level Predictive Capability for Severity of Restenosis Post Percutaneous Coronary Intervention

**DOI:** 10.3389/fphar.2022.816059

**Published:** 2022-05-24

**Authors:** Jiqiang Guo, Ying Gao, Mohammad Ahmed, Pengfei Dong, Yuping Gao, Zhihua Gong, Jinwen Liu, Yajie Mao, Zhijie Yue, Qingli Zheng, Jiansheng Li, Jianrong Rong, Yongnian Zhou, Meiwen An, Linxia Gu, Jin Zhang

**Affiliations:** ^1^ College of Biomedical Engineering, Taiyuan University of Technology, Taiyuan, China; ^2^ Shanxi Bethune Hospital, Shanxi Academy of Medical Sciences, Tongji Shanxi Hospital, Third Hospital of Shanxi Medical University, Taiyuan, China; ^3^ Tongji Hospital, Tongji Medical College, Huazhong University of Science and Technology, Wuhan, China; ^4^ Department of Biomedical and Chemical Engineering and Sciences, Florida Institute of Technology, Melbourne, FL, United States

**Keywords:** homocysteine, in stent restenosis, severity, percutaneous coronary intervention, ROC curve

## Abstract

**Background:** In stent restenosis (ISR) is one of the major complications after stent implantation. Thus, there is a growing interest in identifying a biomarker for the onset of ISR. High levels of serum homocysteine (Hcy) have been associated with the progression of cardiovascular disease. Therefore, the study was carried out to quantify the correlation between serum Hcy and ISR severity. Compared with coronary angiography (CAG), Hcy levels provided a significantly better clinical detection of ISR severity after PCI.

**Methods:** A total of 155 patients were recruited from Shanxi Bethune hospital, from 6 months to 2 years post PCI. Serum Hcy levels and postoperative angiography results were used to differentiate the patients into two experimental groups: ISR (>50% diametrical stenosis), and non-ISR. The non-ISR included two subgroups: intimal hyperplasia (10–50% diametrical stenosis), and recovery (<10% diametrical stenosis). In addition, a group of 80 healthy individuals was used as a negative control. The correlation between homocysteine level and ISR severity t was analyzed for all groups. In addition, the correlation between serum Hcy level and the severity of ISR in the experimental group was analyzed by the Pearson correlation test.

**Results:** The serum Hcy level in the experimental group and control group was determined to be (20.21 ± 11.42) μmol/L and (15.11 ± 10.25) μmol/L respectively. The level of serum Hcy in the experimental group was significantly higher than in the control group (*t*-value of 2.385; *p*-value of 0.019). The serum Hcy level in the restenosis and the intimal hyperplasia group was (25.72 ± 13.71) μmol/L and (17.35 ± 7.70) μmol/L respectively. The serum Hcy level in the restenosis group was significantly higher than in the intimal hyperplasia group (*t*-value of 2.215; *p*-value of 0.033). The level of serum Hcy in the group without a plaque in the stent was (16.30 ± 6.08) μmol/L, whereas in the control group was (15.11 ± 10.25) μmol/L. The no plaque group had a slightly higher serum Hcy level than the control group (*t*-value of 0.634; *p-*value of 0.528). All included patients were divided into four quartiles based on the serum Hcy concentration: quartile 1 (8.90–13.20 μmol/L), quartile 2 (13.30–16.45 μmol/L), quartile 3 (16.60–24.25 μmol/L) and quartile 4 (24.30-65.30 μ mol/L). The incidence of ISR was 5, 6.25, 7.5 and 15%, in the 1,2,3 and four quartiles respectively. The serum Hcy level in the experimental group was (20.21 ± 11.42) μmol/L, the severity of in-stent restenosis was (0.25 ± 0.31), (*R-*value was 0.234; *p-*value was 0.037), indicating a correlation between serum Hcy and the severity of restenosis (*p* < 0.05). Taking coronary angiography as the gold standard, a ROC curve analysis was performed on the serum Hcy levels for the experimental group. The area under the curve (AUC) was 0.718 (95% *CI* 0.585-0.854, *p* < 0.001), indicating that the serum Hcy concentration could predict ISR. On the ROC curve, the best critical value of serum Hcy concentration for predicting ISR was 20.05 μmol/L, with a sensitivity of 45% and specificity of 88.1%.

**Conclusion:** A positive correlation was observed between homocysteine and the severity of restenosis after PCI, The level of Hcy could serve as a predictive biomarker for the severity of ISR.

## 1 Introduction

The continuous improvement in people’s living standard, the change of lifestyle and eating habits have aggregated the incidence rate of coronary heart disease, especially towards the younger ages. Therefore the occurrence and development of coronary heart disease significantly reduces patient’s quality of life. Percutaneous coronary intervention (PCI), also known as stenting, is the popular treatment procedure to open clogged arteries with the advantages of short operation time and minimal invasiveness (Bennett 2003). However, stent restenosis (ISR) is one of the major complications following PCI (Giulio and David 2013). The ISR is a complex pathophysiological process, including vascular inflammation, vascular remodeling caused by endothelial injury, and excessive proliferation and migration of vascular smooth muscle cells. Large-scale clinical trials have shown that the incidence of ISR of bare metal stents is approximately 20–30%. However, drug-eluting stents could reduce the risk of restenosis to 5–10% (Paudel et al., 2005).

A variety of cytokines and inflammatory factors such as nuclear factors-κB (NF-κB), tumor necrosis factor-α (TNF-α), platelet derived growth factor (PDGF) have been associated with the incidence of coronary ISR (Guildford et al., 2011). The high homocysteine (Hcy) level, i.e., hyperhomocysteinemia, has attracted increasing attention in treating coronary heart disease. High Hcy level was positively correlated with the severity of coronary heart disease, atrial fibrillation, stroke, arteriosclerosis, and other cardiovascular disorders (CADS) (Chen et al., 2018; Dhar et al., 2018; Kubota et al., 2019). Hcy is a sulfur-containing amino acid, which can be easily detected in the blood. It is mainly metabolized by methionine (MET) through two main pathways: re-methylation to methionine or reverses sulfurization to cysteine (Kim et al., 2018). Moreover gene mutations encoding enzymes in the Hcy metabolic pathway, an increased homocysteine level can be attributed to vitamin deficiency, excessive methionine intake, or the use of some drugs and other factors (Kumar et al., 2017). However, there is a lack of information regarding the correlation between. Hcy and restenosis. Bakoyiannis et al. (2015) considered Hcy as the risk factor for the efficacy of percutaneous transluminal coronary angioplasty (PTCA). A lower Hcy level could improve the prognosis after PTCA. .De Luca et al., 2005 demonstrated a positive relationship between the Hcy level and the carotid restenosis within 2 years post endarterectomy. Whereas Wong et al., 2004 observed no direct relationship between the level of Hcy and ISI, a higher Hcy level increased the risk of mortality after PCI. However, all these observations were qualitative. Hcy level is important in predicting the disease risk, controlling disease complications and affecting disease outcomes. Hcy level monitoring might be conducive to early detection and diagnosis of diseases, and early prevention of serious complications. However, no quantitative analysis was observed between the Hcy level and the ISR severity. Thus, the study examines the correlation between Hcy and ISR severity, to identify the prediction capacity of Hcy.

## 2 Methods

### 2.1 Patients

In a retrospective study, 155 patients were recruited from Shanxi Bethune hospital as the experimental group 6 months to 2 years following PCI, while 80 healthy individuals were recruited as the negative control group. The clinical data collected in this study was obtained from the inpatient medical record system (patient admission course record), test data system, PCI registration system, and image diagnosis report workstation of the cardiovascular department of Shanxi Bethune hospital. The data included gender, age, serum Hcy levels, and postoperative angiographic results. The clinical data of the experimental group was used to subdivide the group further based on the severity of restenosis after stent implantation. There were 48 cases in the stent restenosis group, 30 cases in the intimal hyperplasia group and 77 cases in the plaque free group. The average age of all patients in the experimental group was (59.89 ± 3.15) years, with 78 male and 77 female patients. The negative control group consisted of 43 males and 37 females ranging from 18 to 60 years of age: the average age of (40.12 ± 4.13 years). The experimental and control groups were comparable for age or sex ratio (*p* > 0.05). All patients had good compliance with the drug treatment after PCI. The patients who successfully received PCI underwent routine coronary angiography in the Shanxi Bethune hospital for recurrent chest distress, chest pain, palpitation, shortness of breath, acid reflux, heartburn, and other symptoms. All procedures were carried out per Shanxi Bethune hospital’s clinical medical ethics standards. The research protocol was approved by the clinical medical ethics committee, and informed consent was obtained from the subjects.

Inclusion Criteria: 1) a complete medical history,2) complaints of stable or unstable angina pectoris leading to further coronary angiography within 6 months to 2 years post stent implantation, and 3) the patient receiving conventional antiplatelet drugs (aspirin 100 mg/Day and clopidogrel 75 mg/Day) and statins for at least 1 year after PCI. The international common visual diameter method was used to calculate the degree of coronary artery stenosis independently, and then the average value was obtained for analysis, (see Eq. 1). Exclusion Criteria:1) taking anti-infective drugs, anti-inflammatory drugs, or glucocorticoids affecting immune function, 2) liver insufficiency, renal insufficiency, impaired right ventricular function, and acute heart failure, 3) recent history of surgery and ulcers, and 4) myocarditis, cardiomyopathy, acute and chronic infection, cardiogenic shock, severe lung disease, severe arrhythmia, blood system disease, peripheral vascular embolism disease, immune system disease, malignant tumor, or severe anemia.

Eq. 1: Degree of stenosis = (Normal vessel diameter near the heart at the stenosis - Vessel diameter at the stenosis)/Vessel diameter near the heart at the stenosis×100%.

### 2.2 Measures

The patient’s gender, age, BMI, coronary heart disease risk factors (hypertension, hyperlipidemia, diabetes, smoking, etc.), relevant laboratory examination indices, coronary angiography characteristics (vascular lesion location), and stent placement conditions (stent diameter, number, length) were collected for the experimental group. Other information includes postoperative medication such as clopidogrel for at least 1 year after stent implantation, aspirin enteric coated tablets, statins *β*- Receptor blockers and angiotensin converting enzyme inhibitors were also obtained.

A total of 155 patients in the experimental group were hospitalized for coronary angiography 6 months to 2 years post PCI. The following day, the elbow venous blood was taken on an empty stomach and sent to the laboratory for hematological examination. Total 5.0 ml of venous blood was collected in a tube containing coagulant separation glue, and the serum Hcy level was detected by the Beckman Kurt AU5800 automatic biochemical analyzer. The cyclic enzyme method was used to determine Hcy levels for the experimental and control group.

All patients underwent coronary angiography in Shanxi Bethune hospital’s cardiovascular interventional catheter room of ISR grouping criteria: related to a plaque in the stent, the patients were further divided into plaque in the stent group and no plaque in the stent group. According to the severity, the plaque in the stent group was divided into the in-stent restenosis and in-stent intimal hyperplasia group. In stent restenosis group: plaques in the stent, and the lumen were lost in the whole process of the stent and/or 5 mm segments at both ends of the stent, resulting in the degree of lumen stenosis ≥50%, and the degree of restenosis can be quantified; In-stent intimal hyperplasia group: plaques in the stent, and the lumen were lost in the whole process of the stent and/or in the 5 mm segment at both ends of the stent, resulting in the stenosis of the lumen ranging from 0 to 50%; No plaque in stent group: no plaque in the whole length of the stent and/or 5 mm segments at both ends of the stent.

### 2.3 Statistical Methods

Excel software was used to establish a database and SPSS 19.0 software was used for statistical analysis. Quantitative data (or measurement data) was described by mean ± standard deviation (‾*x* ± *s*) or median. *t*-test, or analysis of variance, was used for inter-group comparisons. The Pearson method was used for the correlation analysis. Multivariate stepwise logistic regression analysis was used to determine the predictors of ISR. A *p* less than 0.05 was considered statistically significant. The ROC curve was drawn and the area under the curve (AUC) was calculated to determine the accuracy of predicting the Hcy risk. The Youden index method determined the best cutoff point; the maximum sensitivity and specificity determine the critical point. The AUC was compared using medcalc statistical software.

## 3 Results

### 3.1 Clinical Characteristics of Patients

According to the severity of restenosis, or lack thereof, the experimental group was divided into the ISR group (*n* = 48) or the non-ISR group (*n* = 107; subdivided into intimal hyperplasia group *n* = 30 and normal coronary lumen group *n* = 77). No significant difference in gender, average age, smoking, or hypertension was observed between the two groups. Compared with the non-ISR group, the ISR patients had a significantly higher proportion of diabetes mellitus, longer stent implantations, higher serum hs-CRP, HMGB1 concentrations, longer stent lengths, and smaller lumen diameters (Table 1), No correlation was observed between ISR occurrence and age, gender, smoking history, diabetic history, location of the lesion, or hypertension drugs. However, a relationship between ISR and hypertensive history, number of stent implantations, stent length, stent diameter, the diameter of the reference vessel, serum inflammatory factor CRP and HMGB1 levels were observed.

**TABLE 1:** Comparison of serum Hcy levels between non ISR group and ISR group.

Group		Non-ISR group (n = 107)	ISR group (n = 48)	*p* Value
Age (y)		55.23 ± 1.09	57.83 ± 2.16	0.18
Male gender (n, %)		50 (46.4)	28 (58.3)	0.57
Smoking (n, %)		63 (58.9)	30 (62.5)	0.49
Diabetes (n, %)		24.6 ± 3.2	25.5 ± 3.8	0.57
Hypertension (n, %)		86 (80.7)	42 (86.7)	0.046
Target vessels (n, %)	Left anterior descending	50 (46.5)	26 (54.2)	0.61
	Left circumflex	25 (23.2)	14 (29.2)	0.44
	Right coronary artery	31 (28.6)	16 (33.3)	0.42
Stent number (n)		2.10 ± 0.076	1.96 ± 0.068	0.041
Stent length (mm)		19.34 ± 0.5	21.88 ± 0.48	0.026
Stent diameter (mm)		3.13 ± 0.46	2.91 ± 0.54	<0.01
References vessel diameter (mm)		3.05 ± 0.48	2.89 ± 0.43	<0.01
hs-CRP (mg/l)		2.21 ± 1.05	2.77 ± 1.40	0.046
HMGB1 (μg/l)		15.45 ± 7.72	23.95 ± 11.05	0.013
β-blocker (n, %)		21 (37.5)	10 (41.7)	0.62
ACEI/ARB (n, %)		27 (48.2)	13 (54.2)	0.70
CCB (n, %)		29 (51.8)	14 (58.3)	0.35

### 3.2 The Higher Serum Hcy, Levels Revise and Remove Comma

**FIGURE 1:**
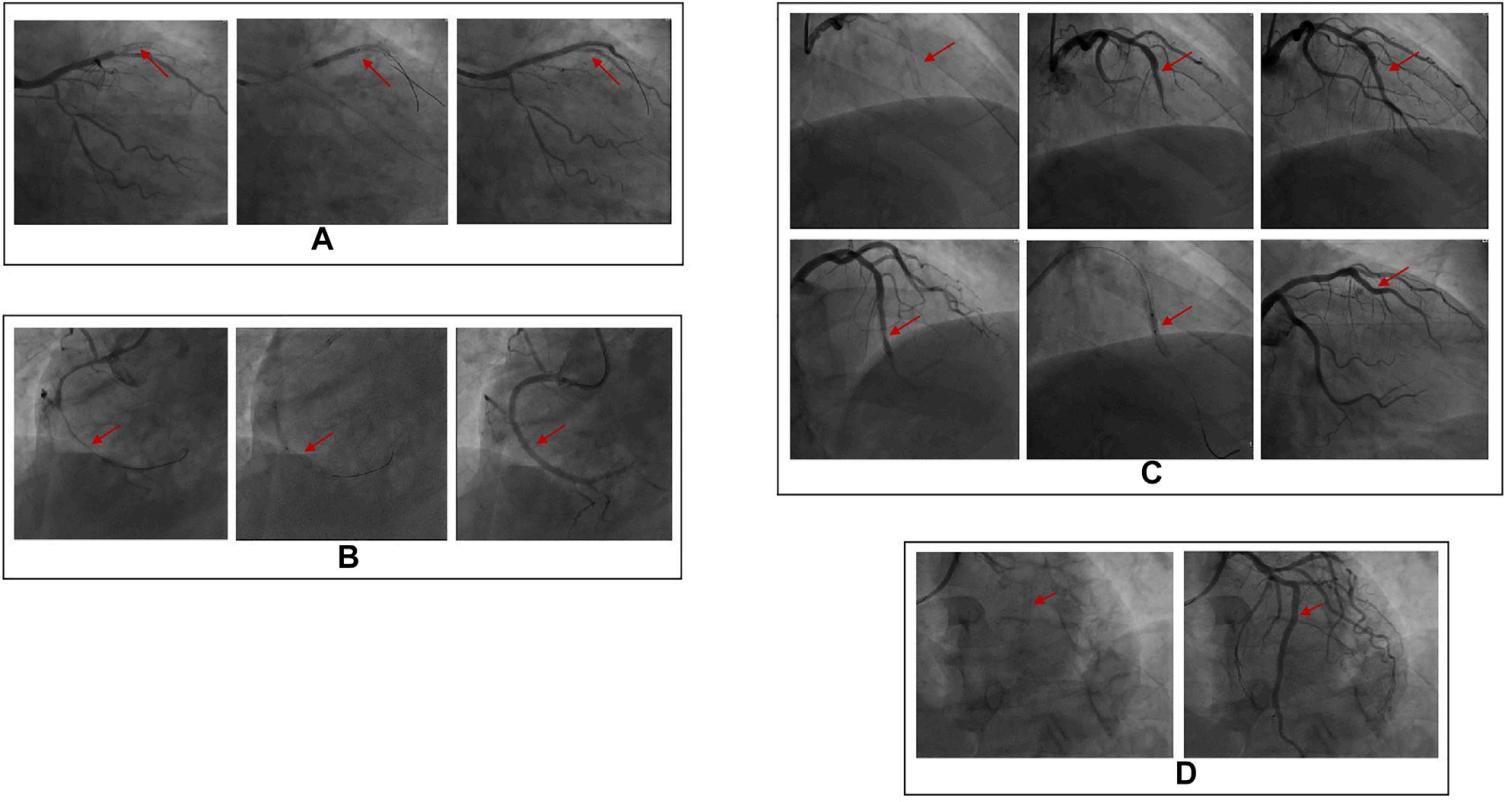
DSA images of plaque formation in stent and the severity of plaque on vascular blockage. **(A)** In stent restenosis (complete occlusion), stent shadow can be seen in the proximal part of lad, complete occlusion in the middle part of LAD (100% stenosis), TIMI grade 0; **(B)** In stent restenosis (partial occlusion), stent shadow can be seen in the proximal part of RCA, and 70% stenosis in the proximal part of RCA; **(C)** There are plaques (intimal hyperplasia) in the stent, stent shadow can be seen in the middle of lad, and intimal hyperplasia in the stent. **(D)** There is no plaque (unobstructed blood flow) in the stent. The stent shadow can be seen in the proximal and middle part of RCA, and the blood flow in the stent is unobstructed.

The clinical data were collected for 155 patients after PCI was collected in the experimental group, while the physical examination data were obtained for 80 healthy individuals was collected in the control group. DSA images of plaque formed in the stent and the degree of vascular blockage caused by plaque in the experimental group are shown in Figure 1. The serum Hcy level in the experimental group was significantly higher than the control group (Table 2) (*t*-value was 2.385, the *p*-value was 0.019). Besides, a correlation was observed between coronary atherosclerosis and Hcy levels. The higher serum Hcy, levels were associated with the increased vulnerability for coronary atherosclerosis. In the experimental group, the serum Hcy level of ISR patients (48 cases) was comparable with the intimal hyperplasia patients (30 cases) (Table 3). The ISR group revealed a serum Hcy level of 25.72 ± 13.71 μmol/L whereas the intimal hyperplasia group had a Hcy level of 17.35 ± 7.70 μmol/L. The difference between the two groups was statistically significant with the *t-*value was 2.215 and the *p*-value was 0.033. Among both the ISR and intimal hyperplasia groups, 77 patients had a serum Hcy level of 22.37 ± 12.28 μmol/L. Patients without a plaque in the stent after PCI revealed an Hcy level of 16.30 ± 6.08 μmol/L. A significant difference was observed between the plaque versus no plaque groups (*t*-value was 2.801 and the *p-*value was 0.006). The plaque free group had a lower Hcy level (Table 4), the higher the serum Hcy level, the greater the probability and severity of ISR. Patients with a plaque in the stent after PCI had an Hcy level of 22.37 ± 12.28 μmol/L whereas patients in the plaque free group had the level of 16.30 ± 6.08 μmol/L. The healthy subjects in the control group had a level of 15.11 ± 10.25 μmol/L, which was further lower than the no plaque group. The difference between the no plaque group and control group was statistically significant (*t*-value was 2.872 and the *p-*value was 0.005). No statistically significant difference was observed in serum Hcy levels of the plaque free group (*t-*value was 0.634 and the *p*-value was 0.528), (Table 5).

**TABLE 2:** Comparison of serum Hcy levels between experimental group and control group.

Variables	Hcy (μmol/L)	Number of Cases(n)	*t* value	*p* value
Experience group	20.21 ± 11.42	155	2.385	0.019
Control group	15.11 ± 10.25	80		

**TABLE 3:** Comparison of serum Hcy levels between restenosis group and intimal hyperplasia group.

Variables	Experience group		Control group		*t* value	*p* value
	Hcy (μmol/L)	Number of Cases(n)	Hcy (μmol/L)	Number of Cases(n)		
Restenosis group	25.72 ± 13.71	48	15.11 ± 10.25	80	3.527	0.001
Intimal hyperplasia group	17.35 ± 7.70	30			0.789	0.434
No plaque in stent group	16.30 ± 6.08	77			0.634	0.528
*t* value	6.784					
*p* value	0.000					

**TABLE 4:** Comparison of serum Hcy levels between plaque group and plaque free group in stent.

Variables	Plaque group in stent		No plaque in stent group		*t* value	*p* value
	Hcy (μmol/L)	Number of Cases(n)	Hcy (μmol/L)	Number of Cases(n)		
Restenosis group	25.72 ± 13.71	48	16.30 ± 6.08	77	3.783	0.000
Intimal hyperplasia group	17.35 ± 7.70	30			0.539	0.592
*t* value	2.215					
*p* value	0.033					

**TABLE 5:** Comparison of serum Hcy levels between plaque free group and control group.

Variables	Plaque group in stent		Control group		*t* value	*p* value
	Hcy (μmol/L)	Number of Cases(n)	Hcy (μmol/L)	Number of Cases(n)		
Plaque group in stent	22.37 ± 12.28	78	15.11 ± 10.25	80	2.872	0.005
No plaque in stent group	16.30 ± 6.08	77			0.634	0.528
*t* value	2.801					
*p* value	0.006					

**FIGURE 2:**
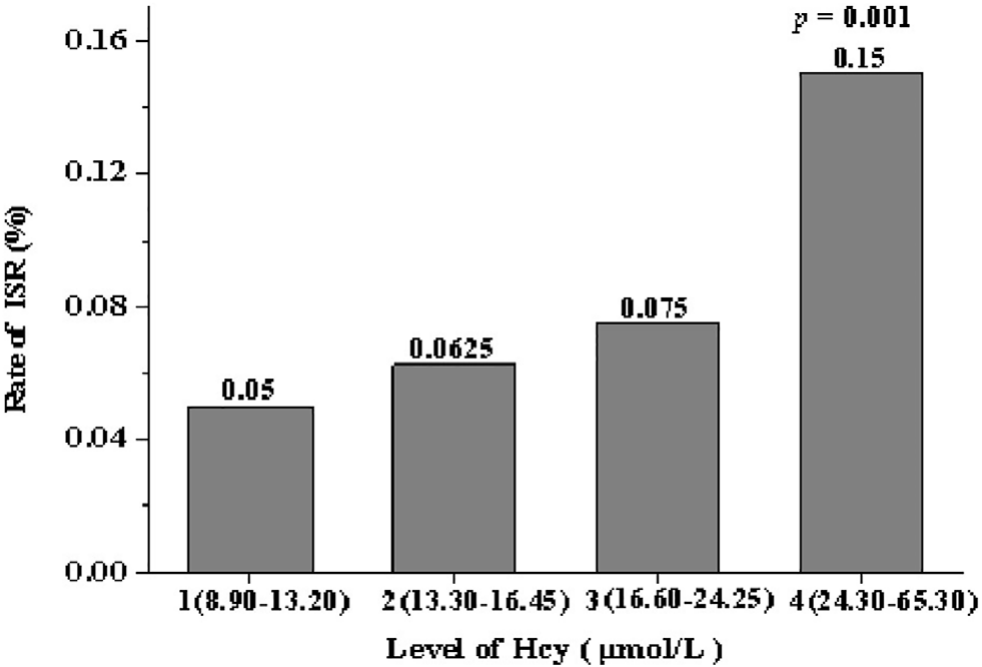
Rate of ISR stratified by quartile of level of Hcy.

The experimental group was further divided into four quartiles (*n* = 20 each quartile) depending on the concentration of serum Hcy: quartile 1 (8.90–13.20 μmol/L), quartile 2 (13.30–16.45 μmol/L), quartile 3 (16.60–24.25 μmol/L) and quartile 4 (24.30–65.30 μmol/L). From quartile one to quartile 4, the incidence of ISR was 5, 6.25, 7.5 and 15%, respectively. The incidence of ISR in patients in quartile four was significantly higher than in other groups (*p* = 0.001), and the incidence of ISR increased gradually between these quartiles (Figure 2).

Of the 155 patients in the experimental group, the severity of ISR in the intimal hyperplasia group was calculated as 0.25, whereas the non-plaque group was 0.00. The correlation between serum Hcy levels and ISR severity was analyzed by the Pearson correlation test (Table 6). The level of serum Hcy in the experimental group was calculated as 20.21 ± 11.42 μmol/L, and the severity of ISR was 0.25 ± 0.31 (*r* = 0.234, *p* = 0.037).

**TABLE 6:** Pearson correlation between serum Hcy value and in stent restenosis severity.

Variables	Detection result	Number of Cases(n)	*r* value	*p* value
Hcy (μmol/L)	20.21 ± 11.42	155	0.266	0.003
In stent restenosis severi (%)	0.25 ± 0.31	155		

**FIGURE 3:**
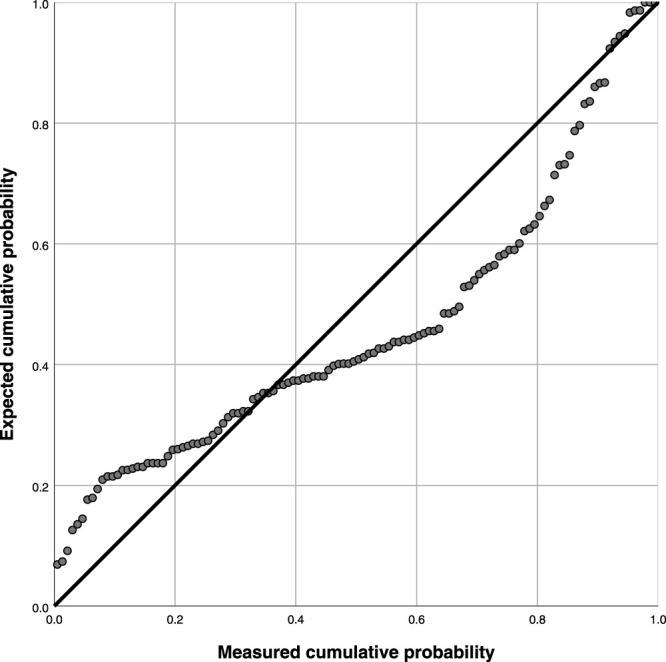
Logistic regression analysis of serum Hcy level and the severity of in stent restenosis.

Table 7 and Figure 3 demonstrate the goodness of fit coefficient (0.266), with multiple linearities. There was significant linearity since a regression coefficient *p* was less than 0.05 indicating a linear relationship (*p* = 0.003). (Linear regression equation: *y* = 16.717 + 10.876*x*, 95% *CI*: 22.19–61.45).

**TABLE 7:** Logistic regression analysis of restenosis severity.

Variables	R	95% CI	*p* value
Serum Hcy	0.266	22.19-61.45	0.003

### 3.3 Correlation Analysis Between the Severity of in Stent Restenosis and Diseased Vessels

A total of 82 patients (52.90%) had vascular lesions in the left anterior descending branch in the experimental group., In the ISR group, the most significant incidence of ISR was related to the left anterior descending coronary artery (LAD) in 26 cases (54.17%), followed by 16 cases (33.33%) in the right coronary artery (RCA), 4 cases (8.33%) in the left circumflex artery (LCX), and 2 cases (4.17%) in the left main coronary artery, (LM) (Table 8).

**TABLE 8:** Pearson correlation between diseased vessels and the severity of in stent restenosis.

Diseased vessel	RCA	LAD	LM	LCX	Total
Restenosis	16 (33.33%)	26 (54.17%)	2 (4.17%)	4 (8.33%)	48 (30.97%)
Intimal hyperplasia	3 (8.57%)	24 (68.57%)	2 (5.72%)	6 (17.14%)	35 (22.58%)
No plaque in stent group	16 (22.22%)	32 (44.44%)	2 (2.78%)	22 (30.56%)	72 (46.45%)
Total	35 (22.58%)	82 (52.90%)	6 (3.87%)	32 (20.65%)	155 (100%)
*X* ^*2*^ value	15.814				
*p* value	0.015				

### 3.4 ROC Curve Analysis Value of Serum Hcy Level in Predicting the Severity of in Stent Restenosis

**FIGURE 4:**
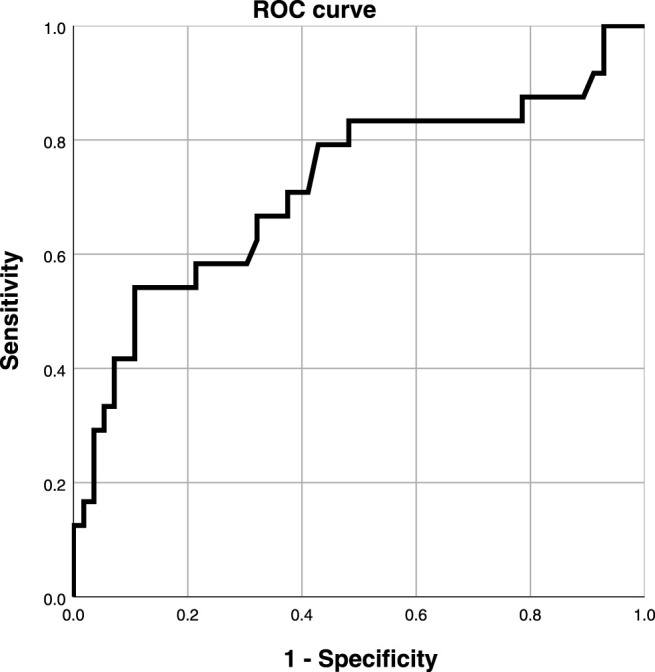
ROC curve of serum Hcy level.

Taking coronary angiography as the gold standard, the ROC curve was used to explore the relationship between the serum Hcy level and ISR in the experimental group (Figure 4). Taking Youden index = sensitivity + specificity - 1, the area under the curve was AUC 0.718 (95% CI 0.585-0.854, *p* < 0.001), indicating that serum Hcy concentration could predict ISR. The best critical value of serum Hcy concentration on the ROC curve for predicting ISR was 20.05 μmol/L with a sensitivity of 45%, and specificity of 88.1%. Compared with other study populations, a serum Hcy value of 20.05 μmol/L significantly increased the risk of restenosis.

## 4 Discussion

The incidence of coronary is increasing amongst younger age groups, as a consequence of dietary changes and increased stress (Assimes 2016). PCI is the popular method to treat coronary heart disease. Though PCI significantly improves the quality-of-life index of patients. ISR is a major adverse event post PCI. The incidence of ISR with bare-metal stents is 20–30%, whereas the emergence of drug-eluting stents reduces ISR incidence to a certain extent, (about 5–10%). Still, drug-eluting stents inhibit intimal hyperplasia and growth of endodermal cells (ECS) while promoting the formation and development of ISR to a certain extent (Tian et al., 2013). The efficacy of biodegradable stents depends on their degradability, however, discontinuity or thrombosis might occur in the degradation process doubling the risk of thrombosis in the stent (Byrne et al., 2015). Current strategies focus on mitigating the ISR, including improving myocardial perfusion, reducing cardiomyocyte necrosis, etc.

During PCI, the balloon expands the stent to enlarge the lumen and push the lesion outwards (Philip 2015). To ensure the complete expansion of the stent and maximum elimination of residual plaque in the blood vessel clinicians often choose to over-expand the stent. Due to the abnormal stress induced by the stent, the ECS in the lesion area experience damage in varying degrees. The ECS injurypromotes the inflammatory cells such as neutrophils and macrophages to start the repair mechanism. This cascade causes an inflammatory response causing the release of inflammatory factors at the damaged part of the vascular endothelium. Under the combined action of many chemokines and inflammatory factors, vascular smooth muscle cells (SMC) proliferate and migrate to the damaged vascular intima, and the abnormal proliferation of vascular smooth muscle cells (Hu et al., 2015) contributes to the formation and development of ISR. In addition, the change in the elastic contraction of blood vessels promotes the occurrence of ISR (Kokkinidis et al., 2019).

Hcy is a sulfur-containing amino acid formed after the demethylation of methionine. Though it is an intermediate product of the methionine cycle, it is an unnecessary amino acid for the human body (Otsuka et al., 2012). Foods with high protein content, such as meat and milk, contain a large amount of methionine which can be transformed into Hcy. Other components in the body, such as vitamins B6, B9, B12, folic acid, betaine, etc catalyzes the conversion of Hcy to glutathione (GSH) and S - adenosylmethionine (SAM). As a result, the serum Hcy positively correlates with methionine concentration in the daily diet.In contrast, a negative correlation of Hcy was observed with the concentration of vitamins B6, B9, B12, folic acid, and betaine in the human body (Ganguly and Alam 2015).

The levels of serum Hcy in the ideal human body are low. The Hcy can be converted into GSH and SAM beneficial to the human body. However, genetic defects, nutrient deficiency, smoking, heavy drinking, and improper diet (excessive intake of foods containing too much lysine or too little intake of vitamins B6, B9, B12, folic acid and betaine) can alter this balance. This imbalance causes an imbalance of nutritional state, resulting in the accumulation of Hcy content and an overall increase in Hcy level in the blood. Studies have shown that elevated serum Hcy can increase the body’s oxidative stress pressure, causeing endothelial cell damage, nitric oxide depletion, reduced endothelial relaxation function, formation of oxidized low-density lipoprotein, and promotion of pre-atherosclerotic state and pre-thrombotic state (Spence 2006; Eichinger 2010; Graeme et al., 2012).

The increase of Hcy levels affects the gene expression of vascular endothelial cells, leading to a toxic effect on the endothelial cells and apoptosis. Therefore, arterial vascular smooth muscle cells overgrow and proliferate, resulting in vascular endothelial wall thickening, arterial elasticity damage and the formation of joint sclerosis plaque in stents. These conditions contribute to be the potential pathogenesis of ISR. However, human serum Hcy level is affected by genetic factors, nutritional and dietary factors, life factors, drug factors, and other factors (Nygrd et al., 1995; Nagele et al., 2011; Naik et al., 2011; Nilsson et al., 2014; Hildebrandt et al., 2015; Jung et al., 2015), These factors include lack of cystine sulfide-β-Synthetase (CBS), methylenetetrahydrofolate reductase (MTHFR), Methionine synthase (MS), methionine adenosyltransferase (MAT) folic acid, vitamin B6, vitamin B12 and othergenetic factors. Besides smoking, drinking, lack of physical exercise and other living habits, antiepileptic drugs, metformin, methotrexate, thiazide diuretics, niacin, rosiglitazone and drugs for hypothyroidism, renal failure, malignant tumor and other diseases, can also increase the blood Hcy level.

De et al. (De Luca et al., 2005) showed that patients with moderate or severe high Hcy levels might have a higher risk of restenosis and subacute thrombosis. Hcy promotes the proliferation of intravascular SMC, inducing SMC to enter the division stage and rapidly proliferate and differentiate in a short period (Zeng et al., 2010); Hcy can also affect the normal metabolism of blood lipids in the human body. Hcy can cause the oxidative modification of low density lipoprotein (LDL), form oxidized low-density lipoprotein (ox LDL), and reduce the concentration of high-density lipoprotein (HDL). In addition, Hcy can reduce NO production and cause damage to vascular function by combining with NO secreted by endothelial cells and ox LDL, leading to limited vascular endothelial relaxation function (Dionisio et al., 2010). High Hcy content can also cause an imbalance of the coagulation and fibrinolysis system. In addition, Hcy activates metalloproteinases, activates inflammatory cells, promotes the production of a variety of inflammatory factors such as monocyte chemoattractant protein, tumor necrosis factor and interleukin family, and promotes neutrophil migration to accelerate the damage to vascular endothelium (Kugler et al., 2015).

Studies have observed that high homocysteine levels increase the risk of restenosis after coronary angioplasty. Elevated homocysteine levels are also known to increase the risk of all-cause mortality, mace and heart death after PCI, suggesting that serum Hcy is a potential risk factor for ISR (Hong et al., 2005). The dose-response meta-analysis supported a linear relationship between homocysteine levels and all-cause mortality in the general population. Specifically, an increase in hcy levels of 5 μmol/l was correlated with the risk of all-cause mortality increase by 1.336 times (Zhang et al., 2019). The alterations of Hcy levels in patients who underwent carotid endarterectomy (CEA) with venous patch closure technique were collected pre-and post-operation in a prospective design. Hcy levels were significantly correlated with both the presence of complicated atheromatous plaque and the degree of internal carotid artery restenosis after CEA (Bakoyiannis et al., 2015; Vidale et al., 2017). However, the relationship between Hcy levels and long-term outcomes post PCI remained inconsistent and contradictory (Stangl et al., 2000; Zairis et al., 2002; Ortolani et al., 2004). Some studies have shown that extending the intake of oral folic acid decreased the plasma Hcy level. Therefore, long-term oral folic acid tablets can reduce the plasma Hcy level and reduce the incidence of coronary stent restenosis. Therefore, folic acid can be used as the secondary prevention of in stent restenosis. Moreover, with its low price and high-cost performance, folic acid can be recommended in the clinic (Schnyder et al., 2001; Schnyder et al., 2002; Hong et al., 2005; Bleie et al., 2008).

There are three main methods of detecting for serum Hcy, circulating enzyme method, isotope method, immunoassay and chromatography. However, the use of these methods is limited in clinical practice due to the involved in operational complexity. In this study, the circulating enzyme method determines the serum Hcy level. The circulating enzyme method uses enzyme-substrate specificity to amplify the target substance. It has the advantages of mild reaction, good specificity, high sensitivity, environmental protection, and no pollution, and it has been widely popularized in clinics (Ortolani et al., 2004).

We have demonstrated that the level of serum Hcy in the experimental and control group was 20.21 ± 11.42 μmol/L and 15.11 ± 10.25 μmol/L, respectively. The serum Hcy in the experimental group was significantly higher than in the control group. Serum Hcy in the groups with plaque in the stent was 22.37 ± 12.28 μmol/L whereas in the plaque free group was 16.30 ± 6.08 μmol/L. The serum Hcy in the plaque group was higher than in the no plaque group. The serum Hcy level was 25.72 ± 13.71 μmol/L in the ISR group and 17.35 ± 7.70 μmol/L in the intimal hyperplasia group. The serum Hcy in the ISR group was higher than in the intimal hyperplasia group. The level of serum Hcy in the group without a plaque in the stent was 16.30 ± 6.08 μmol/L whereas the control group had a level of 15.11 ± 10.25 μmol/L.A small difference was observed in serum Hcy levels between the plaque free group and the control group, indicating a correlation between serum Hcy levels and ISR. Pearson correlation test showed a correlation between serum Hcy value and ISR severity. Using CAG as the gold standard, the ROC curve analysis of serum Hcy level in the experimental group showed that the serum Hcy levels had a certain predictive value for ISR severity.

Overall, a positive correlation was found between serum Hcy level and ISR severity, suggesting that serum Hcy is a risk factor for ISR and a significant predictor of cardiovascular disease. Furthermore, it has a certain predictive value for the formation, development and severity of ISR after PCI. Therefore, early intervention to reduce preoperative and postoperative serum Hcy levels might be helpful to prevent ISR.

## 5 Conclusion

A positive correlation was observed between the serum Hcy level and ISR severity. In addition, the ROC curve analysis demonstrated that the serum Hcy level could serve as a predictive biomarker for ISR severity after PCI. However, other hematological indexes might alter the correlation between serum Hcy and ISR severity, which was not considered in this present study. Future multi-center research with a large cohort is required to validate the current findings in clinical practices.

## Data Availability

The original contributions presented in the study are included in the article/Supplementary Material, further inquiries can be directed to the corresponding authors.
